# Lymphatic Collecting Vessel: New Perspectives on Mechanisms of Contractile Regulation and Potential Lymphatic Contractile Pathways to Target in Obesity and Metabolic Diseases

**DOI:** 10.3389/fphar.2022.848088

**Published:** 2022-03-09

**Authors:** Yang Lee, Scott D. Zawieja, Mariappan Muthuchamy

**Affiliations:** ^1^ Department of Medical Physiology, College of Medicine, Texas A&M University, Bryan, TX, United States; ^2^ Medical Pharmacology and Physiology, School of Medicine, University of Missouri, Columbia, MO, United States

**Keywords:** lymphatic function, lymphatic vessel contraction, lymphatic muscle cells, metabolic disease, sarcoplasmic and myofibrillar proteins

## Abstract

Obesity and metabolic syndrome pose a significant risk for developing cardiovascular disease and remain a critical healthcare challenge. Given the lymphatic system’s role as a nexus for lipid absorption, immune cell trafficking, interstitial fluid and macromolecule homeostasis maintenance, the impact of obesity and metabolic disease on lymphatic function is a burgeoning field in lymphatic research. Work over the past decade has progressed from the association of an obese phenotype with Prox1 haploinsufficiency and the identification of obesity as a risk factor for lymphedema to consistent findings of lymphatic collecting vessel dysfunction across multiple metabolic disease models and organisms and characterization of obesity-induced lymphedema in the morbidly obese. Critically, recent findings have suggested that restoration of lymphatic function can also ameliorate obesity and insulin resistance, positing lymphatic targeted therapies as relevant pharmacological interventions. There remain, however, significant gaps in our understanding of lymphatic collecting vessel function, particularly the mechanisms that regulate the spontaneous contractile activity required for active lymph propulsion and lymph return in humans. In this article, we will review the current findings on lymphatic architecture and collecting vessel function, including recent advances in the ionic basis of lymphatic muscle contractile activity. We will then discuss lymphatic dysfunction observed with metabolic disruption and potential pathways to target with pharmacological approaches to improve lymphatic collecting vessel function.

## Introduction

Obesity afflicts over one-third of the U.S. population, is one of the most critical predictors of metabolic syndrome as well as a significant driver of cardiovascular disease (Friedrich, 2017; Skinner et al., 2018; Garrido-Miguel et al., 2019). The metabolic syndrome is characterized by three or more concurrent metabolic conditions, including hyperglycemia, low high-density lipoprotein (HDL), hypertriglyceridemia, hypertension, and abdominal obesity associated with a greater risk of adverse cardiovascular disease and outcomes (Grundy et al., 2005). Many common rodent models of obesity or diabetes also result in a similar amalgamation of these metabolic perturbations (Wang and Liao, 2012; Lang et al., 2019), although assessing the presence of each of these parameters within the model being employed is often incomplete. Additionally, discrepancies in diet composition (Lang et al., 2019; Bastías-Pérez et al., 2020), diet length (Long et al., 2021), microbiome composition (Long et al., 2021), rodent age, and significant (and often unknown) variability in the genetic composition across mouse strains and substrains (Heiker et al., 2014) are likely to impart significant alterations in disease susceptibility and pathogenesis. This is especially salient with the ubiquitous use of transgenic and/or inducible knockout mouse models that may have incomplete backcrossing or mixed strain composition. It should be noted that mouse strain genetic profiling using SNPs is now commonplace and may help mitigate the substantial effects mouse strain and substrain impart on obesity models (Mekada and Yoshiki, 2021). While a complete profile of each of these variables is untenable, their inclusion will improve our ability to make cross-study observations on metabolic disease-induced lymphatic dysfunction. Despite these challenges, a consistent theme of lymphatic dysfunction, particularly lymphatic collecting vessel contractile dysfunction, has been observed across both diet-induced and genetic models employed in the pathology of metabolic disease over the past decade (Kataru et al., 2020). These findings have augmented our understanding of the serious complications metabolic diseases pose to the cardiovascular system and underlie the need for lymphatic-targeted pharmacological interventions (Jiang et al., 2019; Cao et al., 2021).

The relationship between metabolic disease and lymphatic dysfunction is readily appreciated in the clinical setting, with obesity and diabetes posing increased risk for postoperative lymphedema in cancer patients (Wetzig et al., 2017) and the identification of morbid obesity-induced lymphedema (Fife et al., 2008). While primary lymphedema is due to genetic malformations associated with lymphatic architecture or valve development, the majority of secondary lymphedema cases, not due to filariasis, are typically secondary to cancer therapies, including radiation, node dissection, and chemotherapy (Saaristo et al., 2002; Rockson and Rivera, 2008; Alitalo, 2011; Choi et al., 2012; DiSipio et al., 2013). Excluding the secondary lymphedema resulting from physical damage (which includes cancer-associated lymphadenectomy), obesity, inflammation, and other metabolic diseases are the major contributors of secondary lymphedema (Chakraborty et al., 2010; Greene and Maclellan, 2013; Mehrara and Greene, 2014; Greene et al., 2015; Greene et al., 2020). Lymphedema is a progressive disease characterized by swelling, fibrosis, adipose deposition, and increased risk of infection due to chronic lymphatic dysfunction. Critically, lymphedema patients also exhibit significant impairment in lymphatic collecting vessel contractile regulation with spontaneous contractions either absent, occurring at low frequencies, or with exceptionally weak amplitude (Olszewski, 2002, 2008). The development of obesity-induced lymphedema appears to be largely constrained to morbid obesity, with progressive risk for obesity-induced lymphedema occurring at a BMI over 40 (Greene and Sudduth, 2021) and is almost ubiquitous at BMIs over 60 (Sudduth and Greene, 2021). While weight loss in these patients can be achieved, lymphedema could be irreversible despite significant weight loss (Greene and Sudduth, 2021; Sudduth and Greene, 2021). Interestingly, while noting that lymphatic dysfunction occurs throughout the obesity spectrum (Blum et al., 2014), one study has identified a weight threshold of >40g is associated with more pronounced lymphatic dysfunction using a diet-induced obesity mouse model (Nitti et al., 2016). Lymphatic dysfunction likely contributes to the noted risk for infection (Yuan et al., 2019), sub-clinical peripheral edema, and adipose dysfunction noted in human disease (Jiang et al., 2019). Though perception on how lymphatic dysfunction aggravates metabolic syndrome, and reciprocally, the mechanisms by which metabolic disease impairs lymphatic function have progressed (Kataru et al., 2020), there are still significant knowledge gaps in our understanding of these phenomena that prevent the development of lymphatic-specific pharmacological interventions. To date, pharmacological therapies are still largely absent for either restoring or improving lymphatic contractile function or treating lymphedema. Ketoprofen, a 5-Lipooxygenase and cyclooxygenase inhibitor which showed promise in alleviating lymphatic dysfunction in a rodent model of lymphedema (Nakamura et al., 2009), has shown promise ameliorating the skin changes that occur with lymphedema but without a concurrent reduction in limb volume or bioimpedance (Rockson et al., 2018; Rockson, 2021). Clearly, there is still much work to be done to identify physiologically relevant and pharmacologically accessible targets to improve or restore lymphatic function.

The mechanisms by which obesity may interact with the gene pathways critical to lymphangiogenesis and lymphatic endothelial maintenance have been recently described (Norden and Kume, 2021; Ho and Srinivasan, 2020; Kataru et al., 2020). An examination of the effects of vasoactive molecules on lymphatic function and their respective pharmacological implications has also been recently discussed in the exhaustive review (Russell et al., 2021). In this article, we will review: 1) an introduction describing the links between obesity and metabolic syndrome with lymphatic dysfunction and lymphedema 2) a brief synopsis of the lymphatic vasculature to introduce the lymphatic collecting vessels; 3) our understanding of the necessity of the lymphatic pump in physiology; 4) ionic and molecular mechanisms underlying lymphatic contractions; 5) the role of eNOS in lymphatic collecting vessel function and dysfunction; 6) contractile dysfunction in metabolic disease; 7) potential pharmacological targets.

### Lymphatic System and Architecture

The lymphatic system is critical to fluid movement from the interstitial space of the tissue parenchyma (Jamalian et al., 2017) and its transport as lymph through the lymphatic vasculature by both extrinsic and intrinsic forces (Bridenbaugh et al., 2003; Muthuchamy and Zawieja, 2008). In the majority of tissues in the body, interstitial fluid is generated by low-level filtrate from the microvasculature (Michel et al., 2020) and requires an equivalent volume of fluid, as lymph, return to prevent edema. Seemingly complicating the matter, interstitial pressure has been repeatedly measured to be sub-atmospheric (Clough and Smaje, 1978) and lymph must be transported against a net hydrostatic gradient (von der Weid and Zawieja, 2004; Zawieja, 2009; Castorena-Gonzalez et al., 2018) without the assistance of a centralized pump such as the heart. Nevertheless, the interconnected network of initial lymphatics, collecting lymphatic vessels, and lymph nodes that comprise the lymphatic system transport fluid and other critical contents including macromolecules, lipids/chylomicron, and antigen-presenting cells/immune cells from the interstitial space back to the circulation.

The lymphatic capillaries are composed of a single layer of lymphatic endothelial cells (LECs) without a continuous basement membrane and are tethered to the interstitium through anchoring filaments (Baluk et al., 2007; Norrmen et al., 2011). In lymphatic collecting vessels, lymphatic endothelial junctions link LECs into a continuous monolayer to restrict the transport of macromolecules across the endothelial barrier in a highly regulated manner. Therefore, maintaining the lymphatic endothelial barrier is critical for physiological lymphatic roles, including fluid homeostasis, lipid absorption (Cao et al., 2021; Cifarelli et al., 2021), immune cell regulation (Cromer and Zawieja, 2018), and adipose insulin sensitivity (Cao et al., 2021; Cifarelli et al., 2021). Through the use of immunofluorescence and electron microscopy, two types of lymphatic endothelial junctions have been described by their morphological appearance and apparent function; colloquially termed zipper-like junctions and discontinuous button-like junctions (Baluk et al., 2007; Yao et al., 2012). The button-like junctions are predominantly observed in lymphatic capillaries and are thought to serve as the primary lymphatic valves that permit fluid and particle entry into the lymphatic capillary. The current paradigm for fluid entry into the lymphatic capillary posits elevated interstitial pressure opens the intervening space between the button junctions via the tethering of the LECs to the interstitial matrix (Baluk et al., 2007), although transcellular mechanisms have also been suggested to be involved (Triacca et al., 2017). In contrast to the button-like junctions, the zipper-like junctions are predominantly found in the collecting lymphatic vessels. The paradigms regulating vessel permeability in the blood vasculature appear to be recapitulated in lymphatic endothelial barrier regulation in the lymphatic collecting vessels (Scallan and Huxley, 2010; Scallan et al., 2013). Lymphatic collecting vessel permeability has been documented under metabolic disease conditions, including hyperglycemia (Lee et al., 2018), diabetes (Scallan et al., 2015), and adipose dysfunction (Kuan et al., 2015; Cao et al., 2021). These alterations in lymphatic collecting vessel permeability are unsurprising given the extensive investiture and association of immune cells within the lymphatic collecting vessels (Bridenbaugh et al., 2013; Chakraborty et al., 2015; Zawieja et al., 2016b; Ivanov et al., 2016; Randolph et al., 2016; Cromer and Zawieja, 2018) and the role inflammatory cytokines play in regulating lymphatic endothelial barrier integrity (Cromer et al., 2014). It should be noted that both button and zipper junctions are observed in the lymphatic capillaries found in the villi of the intestine, termed lacteals, and these junctions are dynamically regulated for proper lipoprotein uptake and normal fat absorption (Bernier-Latmani and Petrova, 2017; Zhang et al., 2018). Targeting the intestinal lacteals (Bernier-Latmani et al., 2015; Nurmi et al., 2015) or the LEC-LEC junctions (Zhang et al., 2018) in the lacteal hold promise as potential pharmacological targets in obesity. LEC junction regulation in metabolic disease has been reviewed elsewhere (Zhang et al., 2018; Norden and Kume, 2021; Kataru et al., 2020).

In the vast majority of vascular beds, interstitial fluid is constantly formed from a low-level capillary filtration required to support the steady state oncotic pressure difference across the vascular wall (Levick and Michel, 2010; Michel et al., 2020). The mechanism(s), and the necessary forces they must generate, to regulate lymph formation under physiological conditions are still being queried. Once in the lymphatic capillary, lymph is then moved towards the larger collecting lymphatic vessels that consist of LECs basement membrane and are surrounded by lymphatic muscle cells (LMCs) (Muthuchamy et al., 2003; Petrova et al., 2004; Muthuchamy and Zawieja, 2008). The LMCs contain a unique combination of striated and smooth muscle components that enable the rapid tonic and phasic contractions of lymphatic vessels (Muthuchamy et al., 2003; Muthuchamy and Zawieja, 2008). These lymphatic contractions may also promote lymph formation in the upstream capillaries that exist in tissues with sub-atmospheric pressure. Jamalian et al., 2017 provided evidence for a suction effect that could be transmitted upstream during the relaxation phase of the lymphatic collecting vessel contraction (Jamalian et al., 2017). After passing through multiple chains of lymph nodes and larger collecting lymphatic vessels, the lymph is eventually transported to the thoracic duct that merges into the blood circulation via the left subclavian veins. In addition, lymph fluid from the right side of the body above the diaphragm, including the right section of the trunk, right arm, and right side of the head and neck, will be drained by the right jugular duct (Zawieja, 2009).

### Physiology of the Lymphatic Pump and Importance of LMCs in Lymphatic Contraction

The lymphatic system utilizes energy for propelling lymph flow through the lymphatic network via unique lymphatic pumping mechanisms (Hargens and Zweifach, 1977; von der Weid and Zawieja, 2004; Muthuchamy and Zawieja, 2008; Zawieja, 2009). Several motive forces generate lymph flow centrally with functional lymphatic secondary valves between lymphangions (Mislin, 1976; Zawieja et al., 1993; Zawieja, 1996). Based on the source of energy, the forces that regulate lymphatic pumping can be divided into two categories: 1) the intrinsic or active lymph force and 2) the extrinsic or passive lymph pump. The intrinsic or active pumps are critical for lymph flow in most lymphatic beds, yet mammals do not possess the lymph hearts as lower vertebrates species for pumping the lymph. Instead, the coordinated contractions of LMCs within the lymphangion act as pumps that generate the lymph flow (Muthuchamy et al., 2003; Muthuchamy and Zawieja, 2008) and this contractile activity is heavily dependent on pressure (McHale and Roddie, 1976; Hargens and Zweifach, 1977).

Lymphatic contraction is regulated by combinations of various autonomous and paracrine factors, including the surrounding microenvironment of lymphatic vessels and adjacent tissue beds (Mislin, 1976; Olszewski and Engeset, 1979; Bridenbaugh et al., 2003; von der Weid and Zawieja, 2004), and likely responsive to the contents of lymph itself, especially under pathophysiological states. Thus, like the smooth muscle vasculature of the blood circulation, the lymphatic muscle cells respond to both physical, biochemical, and immunological signals to regulate lymph output from their draining tissue. Expectedly, this tissue specificity is recapitulated in the contractile behavior displayed by lymphatic collecting vessels studied from separate tissues when exposed to the same stimuli (Gashev et al., 2002; Zawieja et al., 2018) and with significant morphological and contractile differences noted across species (Scallan et al., 2016). Conceptually, the field has organized this spectrum of contractile activity into two broad teleological categories, including a “pump” behavior noted in the smaller and pre-nodal vessels and a ‘conduit behavior in the larger lymphatic ducts (Quick et al., 2009). There is evidence for metabolic disease impairing both of these behaviors (Zawieja et al., 2012; Blum et al., 2014; Zawieja et al., 2016a). Lymphatic collecting vessels exhibit rhythmic, fast phasic contractions that drive lymph flow largely as a consequence of generating the pressure stimuli necessary to open the downstream valve and expel the lymph into the next lymphangion. Additionally, lymphatic collecting vessels display lymphatic tonic contraction, and in some cases myogenic constriction (Zawieja et al., 2018), which can also be modulated by lymph flow associated with local, neural, and humoral factors, including α-adrenergic agonist, prostaglandins, bradykinin, substance p (Bridenbaugh et al., 2003; von der Weid and Zawieja, 2004; Muthuchamy and Zawieja, 2008; Russell et al., 2021). These factors modulate lymphatic collecting vessel tone and thus alter resistance to lymph flow to promote or impede lymph flow from the upstream lymphatic bed (Davis et al., 2008; Dougherty et al., 2008; Muthuchamy and Zawieja, 2008; von der Weid and Muthuchamy, 2010; Davis et al., 2012). More recently, quantitative assessments of lymphatic secondary valve function have also shown the importance of lymphatic vessel diameter, and thus lymphatic tone regulation, as the pressure differential required to close the lymphatic valve is seemingly affected by not only lymphatic valve leaflets but also the diameter of the lymphatic vessel (Bertram et al., 2014; Bertram et al., 2017; Lapinski et al., 2017). Lymphatic valves can become incompetent in human lymphedema (Olszewski, 2002); however, whether lymphatic valve function is also impaired in obesity is currently being assessed. Recent findings (Niimi et al., 2021; Scallan et al., 2021) have unveiled a role for FoxO1 as a repressor of lymphatic valve formation, and its inhibition or deletion could be a therapeutic option to increase both the number of valves and possibly the function of existing lymphatic valves.

During the intrinsic phasic contraction, LMCs generate coordinated contraction, mediated by electrical coupling of the lymphatic muscle cells by connexins (Zawieja et al., 1993; Castorena-Gonzalez et al., 2018; Hald et al., 2018), that modulates lymph flow through a lymphatic bed (Mislin, 1976; Olszewski and Engeset, 1979; McHale and Meharg, 1992; Zawieja et al., 1993; Zawieja, 1996). The unique lymphatic contractile characteristic results from the special feature of LMCs that comprise both smooth and striated muscle regulatory mechanisms to accomplish essential functions in the maintenance of the lymph flow (Muthuchamy et al., 2003).

### Molecular Mechanisms of Lymphatic Contraction

a) Role of membrane potential and L-type dependent Ca^2+^ influx in lymphatic contractility

Similar to cardiac or classical smooth muscle, LMCs are excitable cells with each contraction driven by an action potential (AP) (Kirkpatrick and McHale, 1977; Van Helden, 1993). Thus far, APs have been recorded from lymphatic collecting vessels isolated from a host species including human (Telinius et al., 2014b), sheep (Beckett et al., 2007), bovine (Azuma et al., 1977; McHale et al., 1980), mouse (Zawieja et al., 2018; Zawieja et al., 2019; To et al., 2020), rat (von der Weid et al., 2014), and guinea pig (Van Helden, 1993; von der Weid, 1998). Despite appreciable differences in AP shape (suggesting significant differences in the symphony of ion channels involved), AP duration, frequency, and resting membrane potential across these species, L-type voltage-gated Ca^2+^ channels remain fundamentally necessary for lymphatic collecting vessel APs and contractions (Cotton et al., 1997; Hollywood et al., 1997; von der Weid and Van Helden, 1997; Beckett et al., 2007; Lee et al., 2014; Telinius et al., 2014b; Zawieja et al., 2018; To et al., 2020). Lymphatic contractions cease upon maximal L-type calcium channel inhibition, oftentimes complicating pharmacological investigations into the contributions of other ion channels using pharmacological tools due to frequent off-target side effects on L-type calcium channels (Boedtkjer et al., 2015). In mice, the LMCs appear to express the smooth muscle Cav1.2 isoform (with Exon1b), and deletion of Cav1.2 using a smooth muscle-specific Cre (MHC11CreERT2) stops murine lymphatic vessel contractions (To et al., 2020). Intriguingly, Telinius et al. (2014b) reported expression of the cardiac Cav1.2 splice isoform exon 8a in the human thoracic duct (although not confirmed to be LMC specific) (Telinius et al., 2014b), a finding also supported, albeit inconsistently, in murine lymphatic collecting vessels (To et al., 2020). Nonetheless, sub-maximal concentrations of L-type blockers consistently reduce contraction amplitude and force production by the lymphatic muscle (Lee et al., 2014; To et al., 2020). L-type calcium channel blockers are used pharmacologically to treat hypertension, but the concentrations achieved do not seem to impair lymphatic contractions *in vivo* (Telinius et al., 2014b). Conversely, the L-type dihydropyridine channel agonist BayK8644, which can increase the open probability of the L-type channel (Nowycky et al., 1985), significantly increases lymphatic contraction amplitude. A slight reduction in murine collecting vessel contraction frequency is also observed with BayK8644 stimulation (Zawieja et al., 2018; To et al., 2020), likely as a consequence of elongating the AP plateau and a concomitant increase in the duration of the calcium transient associated with the AP in the mouse (Zawieja et al., 2018; Zawieja et al., 2019). L-type calcium channel inhibition with nifedipine also consistently depolarizes LMCs (Van Helden, 1993; von der Weid et al., 2008; Telinius et al., 2014b). Thus, a reduction in LMC L-type calcium channel activity under metabolic disease states may manifest as decreased contraction amplitude and could be accompanied by a slight increase in contraction frequency in rodents but perhaps not humans (Telinius et al., 2014b; To et al., 2020).

Owing to the difficulty in isolating LMCs and recording L-type currents, many questions remain regarding the regulation of L-type calcium channels and their post-translational modifications signaling and scaffolding partners in LMCs under both physiological conditions and in the metabolic disease state. In arterial vascular smooth muscle, high glucose or high-fat diet has been shown to increase L-type calcium channel activity directly (Navedo et al., 2010) and indirectly through the dysregulation of potassium channels and thereby the resting membrane potential (Rainbow et al., 2006; Borbouse et al., 2009; Straub et al., 2009; Nystoriak et al., 2014; Nieves-Cintrón et al., 2016; Greenstein et al., 2020). Work by Nystoriak et al., 2017 demonstrated a direct increase in L-type calcium channel activity contributes to increased myogenic tone and hypertension in response to either acute high glucose or high-fat diet-induced obesity. Increased L-type calcium channel activity and arterial hypercontractility were mediated by PKA-dependent phosphorylation of the pore-forming subunit α1c at Ser 1928 (Nystoriak et al., 2017), which required the scaffolding molecule AKAP150. Whether obesity and metabolic syndrome might affect L-type currents in LMCs has yet to be thoroughly addressed. Further complicating the issue, Cav1.2 involvement in lymphatic collecting vessel tone and frequency may be species and/or vessel bed dependent (Muthuchamy and Zawieja, 2008; Telinius et al., 2014b; Lee et al., 2014; Zawieja et al., 2018; To et al., 2020). Lymphatic collecting vessel tone was not significantly affected in murine popliteal vessels exposed to nifedipine or BayK8644 (To et al., 2020), but lymphatic popliteal collecting vessels from smooth muscle specific Cav1.2KO mice had a significant increase in vessel tone compared to controls (To et al., 2020) Curiously, while 1uM nifedipine does not significantly lower murine popliteal lymphatic vessel tone (To et al., 2020), excessive hyperpolarization of murine LMCs with either KATP channel agonist pinacidil (Davis et al., 2020) or the BK channel activator, NS11021 (Kim et al., 2021) results in significant dilation. These findings, in addition to the elevated murine popliteal lymphatic vessel tone noted in smooth muscle Cav1.2 KO mice, raise interesting questions regarding the role of L-type calcium channels and membrane potential regulation in setting murine lymphatic vessel tone. Targeting L-type calcium channels to improve lymphatic collecting vessel contraction amplitude and function in metabolic disease is seemingly constrained by the potential exacerbation of hypertension in the arterial vasculature, especially in light of the dichotomous findings of nifedipine *in vivo* by Telinius et al., 2014b. Identifying differences in scaffolding or adapter proteins that associate with Cav1.2 or Cav1.2 regulation by phosphorylation and kinases between the arterial smooth muscle cells and LMCs may provide some specific mechanisms for L-type calcium channel targeting with pharmacological approaches.

b) Role of chloride and the calcium-activated chloride channel Ano1 in lymphatic muscle pacemaking

The elongation of the AP duration in murine lymphatic collecting vessels with BayK8644 is seemingly due to L-type calcium influx either directly or indirectly (such as with calcium-induced calcium release) activating the calcium-activated chloride channel, Anoctamin 1 (Ano1) (Toland et al., 2000; Zawieja et al., 2018; Zawieja et al., 2019). The deletion of Ano1 from murine or LMCs or the inhibition of Ano1 pharmacologically significantly altered the murine lymphatic AP, resulting in an action potential characterized by a significantly higher peak membrane potential and an abrogated plateau phase compared to wildtypes (Zawieja et al., 2019). The role of a calcium-activated chloride channel in lymphatic muscle has been appreciated for decades (Van Helden, 1993; Toland et al., 2000; Beckett et al., 2007). Ano1 (also referred to as TMEM16a) was identified as the major calcium-activated channel in human lymphatic thoracic duct and was critically involved in the regulation of spontaneous and alpha-adrenoreceptor contractility (Mohanakumar et al., 2018). The importance of Ano1 in lymphatic pacemaking regulation was further supported by the use of multiple murine models of inducible smooth muscle-specific deletion of Ano1 to specifically target Ano1 in the LMCs (Zawieja et al., 2019). In mice, the reduction in contraction frequency was due to a reduction in the recorded ‘diastolic depolarization’ slope apparent in the mouse recordings (Zawieja et al., 2018; Zawieja et al., 2019), although this diastolic depolarization slope was not significantly elevated when 100 nM BayK8644 was present. Thus, it is tempting to speculate that Ano1, at least in murine lymphatic collecting vessels, may be separately activated by calcium from voltage-gated L-type calcium channels, as likely occurs during the plateau phase of the AP, and that transient subcellular calcium sources seem to activate Ano1 and drive the spontaneous transient inward currents that underlie spontaneous transient depolarizations and the diastolic depolarization phase. As Ano1 is necessary for normal murine lymphatic collecting vessel pacemaking (Zawieja et al., 2019), its expression and activation in LMCs under conditions of metabolic syndrome are of significant interest given the consistency in which impaired lymphatic contraction frequency has been described (Zawieja et al., 2012; Blum et al., 2014; Garcia Nores et al., 2016). In the blood vasculature, Ano1 has also been linked to the hypertensive phenotype associated with high-fat feeding. Leo et al., 2021 observed an increase in Ano1 mRNA expression, protein, and Cl^−^ current density in hindlimb arterial muscle cells in mice fed a high-fat diet for 18 weeks and given streptozotocin in week 14 to mimic the human Type 2 diabetic human phenotype (Leo et al., 2021). Similar investigations into changes in Ano1 expression, regulation of the calcium stores that activate Ano1, and chloride currents in LMCs in models of metabolic syndrome are required. The targeting of Ano1 as a hypertensive therapy could have undesirable effects not only on lymphatic collecting vessel pacemaking activity but also on the regulation of smooth muscle organs that rely on cKit + interstitial cells of Cajal, in which require Ano1 is required for normal slow-wave activity and smooth muscle coordination (Zhu et al., 2009; Cobine et al., 2017; Drumm et al., 2021). However, increasing Ano1 activity to increase lymphatic contraction frequency could also exacerbate hypertension.

Either direct inhibition of Ano1 (Mohanakumar et al., 2018; Zawieja et al., 2019) or alteration of the intracellular chloride concentration (and thus the chloride reversal potential), driven by sodium-potassium-chloride cotransporter (NKCC1) in LMCs, results in hyperpolarization of the LMCs and depressed contraction frequency (Mohanakumar et al., 2018). Mohanakumar et al., 2018 also highlight a clinically relevant consideration for the use of the loop diuretic furosemide (with similar results expected for bumetanide), which inhibits both the kidney-specific NKCC2 and NKCC1 equally and is shown to impair human thoracic duct contractility (Mohanakumar et al., 2018). These findings have been recapitulated in the rat renal lymphatic collecting vessels where NKCC1 inhibition with furosemide decreased bothlymphaticc collecting vessel tone and contraction frequency (Shelton et al., 2020). Inhibition of NKCC1 has been repeatedly demonstrated to lower arterial tone by reducing vascular smooth muscle intracellular chlroide accumulation, thus lowering the chloride reversal potential and hyperpolarizing the muscle cell (Bulley and Jaggar, 2014; Orlov et al., 2015). Hyperpolarization was also observed in the human thoracic duct when chlroide free solutions were applied (Mohanakumar et al., 2018). Inhibition of NKCC1 is being considered as a potential hypertension therapy (Orlov et al., 2015), although how clinically relevant furosemide concentrations affect lymphatic contractile activity *in vivo* must be further evaluated.

c) Role of membrane potential regulation by potassium channels

Potassium channels are critical to setting resting membrane potential in vascular smooth muscle cells. In this role, potassium channels regulate activation of voltage gated calcium channels and arterial contraction (Tykocki et al., 2017). Five major classes of potassium channels have been identified in vascular muscle cells and there is evidence that at least four of these classes represented in LMCs across multiple species (Telinius et al., 2014a; Russell et al., 2021). These include calcium-activated potassium channels (BK_Ca_, Ik_Ca_, Sk_Ca_), inward rectifiers (K_ir_ and K_ATP_), and voltage gated potassium channels (K_V_). Direct evidence for the two-pore domain potassium channels (K2P) has not been determined in lymphatic muscle. K_Ca_, K_v_, and K_ir_ have each been identified in lymphatic muscle through the use of pharmacological inhibitors of varying specificities or potassium substitution, although the specific isoforms and their respective functions has not been fully elucidated as of yet (Allen and McHale, 1988; Cotton et al., 1997; von der Weid and Van Helden, 1997; Beckett et al., 2007; Telinius et al., 2014a).

The limited data within the lymphatic literature detailing potassium channels in LMC regulations is likely constrained by the necessity of sophisticated patch clamp approaches using freshly isolated LMCs in combination with novel genetic knockout approaches to supplement isobaric or isometric data. The first LMC patch clamp study was performed using LMCs isolated from sheep mesenteric vessels (Cotton et al., 1997), in part due to the accessibility of more LMCs compared to rodent vessels, which identified BK channels as the dominant source of the calcium-activated outward current using BK specific inhibitors, Penitrem A and iberiotoxin. Evidence for BK channels has also been documented in human thoracic ducts (Telinius et al., 2014a), in sheep mesenteric LMCs (Beckett et al., 2007), and recently in murine lymphatic LMCs (Kim et al., 2021). Of note, the BK channel inhibitor paxilline has been demonstrated to potently inhibit 90% of calcium-activated chloride current, while iberiotoxin and Pentirem A each reduced calcium-activated chloride current by 20% (Sones et al., 2009). In human thoracic duct isometric preparations, BK inhibition with paxilline increased AP frequency but not amplitude or tone (Telinius et al., 2014a). Similarly, BK inhibition with either iberiotoxin or Penitrem A in mouse popliteal vessels increased contraction frequency, but not amplitude or tone (Kim et al., 2021). In contrast, BK inhibition with Penitrem A in the sheep mesentery decreased frequency without a significant change in resting membrane potential, significantly increased AP amplitude and duration, and was suggested to play a role in early repolarization in the AP. In arterial muscle, BK channels are typically activated by ‘calcium sparks’ which are dependent on ryanodine receptors (RYR) resulting in spontaneous transient outward currents (STOCs) that hyperpolarize vascular smooth muscle and oppose constriction (Nelson et al., 1995). Additionally, calcium sparks and STOCs increase in arterial muscle cells when pressure is raised (Khavandi et al., 2016) and act as a brake on myogenic constriction. This mechanism is dysregulated in high-fat diet induced hypertension (Greenstein et al., 2020). However, a similar role for the RYR-Spark-BK-STOC paradigm in LMCs has yet to be thoroughly addressed.

BK channels have been implicated in the vasodilatory actions of nitric oxide in guinea pig mesenteric vessels (von der Weid and Van Helden, 1997) and recently in murine popliteal collecting vessels (Kim et al., 2021). This suggests that BK channels may play a role in mediating the NO-dependent lymphatic contractile dysfunction observed in obese and metabolic syndrome disease models. The current data regarding the role of K_v_ or K_ir_ channels in lymphatic muscle remains limited due to the use of the broad potassium channel blocker tetraethylammonium (TEA), the non-selective Kv channel inhibitor 4-AP, and barium chloride used to inhibit Kir. Telnius et al. (2014a) provided a critical assessment of the potassium channels present in their isometric human thoracic duct preparation. They identified mRNA for K_v_1.2 (KCNA2), BKCa (KCNMA1), and K_ir_2.1 (KCNJ2) as well as the KATP pore forming subunits K_ir_6.1 and K_ir_6.2. Given the significant contractile differences observed across tissues beds, it seems likely that this is not a complete profile of all the potassium channels expressed in human LMCs. Neither has an exhaustive assessment of K+ channels been performed in mouse or other common rodent models. K_ir_ channels appear poised to regulate resting membrane potential as increasing external potassium to 10 mM hyperpolarized humans LMCs (Telinius et al., 2014a) and inhibiting K_ir_ with barium depolarized LMC resting membrane potential and increased contraction frequency. K_v_ channels also appear to be involved in the repolarization phase of the AP and inhibition with 4-AP consistently drove an acute increase in contraction frequency and amplitude (Telinius et al., 2014a). Whether murine LMCs express a similar profile of K_V_ and K_ir_ channels has yet to be determined, but their roles in regulating both contraction frequency and amplitude may offer hope for therapeutic targeting to restore lymphatic contractility. However, therapeutic goals for restoring lymphatic contractility in metabolic disease will undoubtedly be complicated by the fact that excessive arterial contractility is driving the hypertensive conditions associated with metabolic disease. Downregulation of Kv2.1 (KCNJ2) (Nieves-Cintrón et al., 2016) channel activity has been identified in high-fat fed obesity murine models resulting in increased arterial tone and contractility. Thus, lymphatic specific targeting of K^+^ channels may run the risk promoting or exacerbating hypertension in the patient.

d) Role of SR calcium handling proteins in lymphatic contractility

Using fluorescent calcium dyes (Fluo4, Fura2) (von der Weid et al., 2008; Jo et al., 2019; Stolarz et al., 2019) or endogenously expressed calcium sensors (GCaMPs, RCAMPs) (Castorena-Gonzalez et al., 2018; Zawieja et al., 2019), the global calcium events in lymphatic muscle via L-type calcium channels have been imaged. In addition to the global calcium flashes associated with the APs, spontaneous subcellular calcium transients have been observed during the diastolic period in lymphatic muscle (von der Weid et al., 2008; Castorena-Gonzalez et al., 2018; Zawieja et al., 2019). These calcium ‘puffs’ have been hypothesized to be the calcium source coupled to Ano1 activation and its role in determining LMC excitability and pacemaking (Van Helden, 1993; Imtiaz et al., 2007; von der Weid et al., 2008). The current prevailing hypothesis is that inositol triphosphate receptors mediate calcium efflux from the sarcoendoplasmic reticulum (SR), as vessel contraction or spontaneous transient depolarizations were not affected by ryanodine application in guinea pig mesenteric lymphatic vessels (von der Weid et al., 2008). Conservation of such a seemingly central signaling pathway across mammalian species seems likely, and more work identifying the specific inositol trisphosphate receptor (IP3R) in mouse and human lymphatic collecting vessels is required. However, recent work has demonstrated that rat LMCs also express functional ryanodine receptors (RYR) (Jo et al., 2019; Stolarz et al., 2019; Van et al., 2021) although the expression and role of RYR1 and RYR2 in LMCs remains controversial. Critically, doxorubicin, a chemotherapeutic drug has been linked to lymphedema (Stolarz et al., 2019), impairs lymphatic pumping in a ryanodine receptor-dependent mechanism and this effect is antagonized by the RYR1/3 inhibitor dantrolene (Van et al., 2021). Doxorubicin appears to activate calcium leak from the RYR expressed in the LMCs, and a similar inhibition and calcium leak effect can be achieved with low concentration ryanodine which itself can induce a sub-conductance state and allow calcium to leak from the SR (Jo et al., 2019). The cessation of contractions in response to ryanodine is likely due to sustained depolarization by calcium overload in the cytosol, potentially activating Ano1 or other calcium-activated inward currents and thus preventing the necessary repolarization required to relieve inhibition of L-type calcium channels for oscillatory behavior. Whether doxorubicin, which has profound oxidative properties, is oxidizing ryanodine receptors (or which isoform of ryanodine receptors) or other critical channels such as voltage gated calcium channels or K_ATP_ channels remains unresolved. Increased ROS production is commonly associated with vascular dysfunction in obesity and metabolic syndrome. Aside from the reduction of NO bioavailability, ROS produced in LMCs or LECs of lymphatic collecting vessels could result in RYR oxidation and calcium leak from the SR. Lastly, dantrolene’s inhibitory effect is specific to RYR1 and RYR3, hence its use in malignant hyperthermia. However there is evidence in the cardiac literature that dantrolene may stabilize oxidized ryanodine two receptors and prevent aberrant calcium release (Oda et al., 2015). Whether oxidized RYRs are playing a role in lymphatic collecting vessel dysfunction in metabolic syndrome and can be ameliorated pharmacologically with dantrolene is worth assessing. Genetic manipulation and/or knockout strategies of both specific RYRS and IP3Rs will likely be required to achieve more clarity on this situation.

Recent work in the blood vasculature has reignited interest in the mechanisms by which nitric oxide can cause vessel dilation (Ali et al., 2021). In 2000, Schlossmann et al. identified an IP3R-associated cGMP kinase substrate (IRAG) (Schlossmann et al., 2000) as a mechanism by which NO-cGMP-PKG signaling could inhibit IP3R mediated calcium release. The role of NO-cGMP-PKG-phosphoRAG in mediating vasodilation (Desch et al., 2010) was recently confirmed and explained in more detail by Ali et al., 2021, as TRPM4 transient inward currents were abolished by NO, except when IRAG was knocked down using silencing morpholinos. While TRPM4 has yet to be identified in LMCs, investigations into IRAG modulating IP3-mediated calcium releases in LMCs is warranted, particularly as it pertains to activation of Ano1 and regulation of lymphatic contraction frequency. Lastly, the SR store refilling by SERCA in LMCs (Lee et al., 2020) and the calcium sources utilized to refill the store remain underappreciated in lymphatic muscle. Questions remain as to whether the components of the store operated calcium entry, Stim and Orai, are expressed and if they are functional as would be expected for calcium homeostatic control with frequent SR calcium release (Emrich et al., 2021).

e) Role of myofilament proteins in lymphatic contractility

While intracellular calcium has been considered a key contractile regulator, the role of calcium in the context of lymphatic contractile filament regulation for both basal vessel tone and contraction amplitude is still not fully understood. Understanding this regulation appears to be of significant importance, as lymphatic collecting vessels from lymphedema patients are characterized as dilated and flaccid vessels with poor contraction amplitude (Olszewski and Engeset, 1979). This, in addition to the absence or reduction in frequency of contractions in lymphedema patients, suggests impairment in the LMCs contractile machinery regulation or contractile phenotype maintenance. In thick filament regulation of smooth muscle, increased Ca^2+^ binds to calmodulin, and the Ca^2+^-calmodulin complex then activates myosin light chain kinase (MLCK). MCLK then phosphorylates regulatory myosin light chain 20 (MLC_20_), which activates myosin ATPase and the interaction of myosin with actin. Therefore, MLC_20_ phosphorylation promotes muscle contraction. In contrast, decreased intracellular calcium inactivates MLCK, thus lowering MLC_20_ phosphorylation levels by myosin light chain phosphatase (MLCP) and promoting vessel relaxation. Our laboratory previously found that MLC_20_ phosphorylation is a key component for both lymphatic vessel phasic and tonic contractions (Wang et al., 2009; Nepiyushchikh et al., 2011). Our data indicated two important points: 1) the status of the mono- and di-phosphorylation forms of MLC_20_ affects both tonic and phasic components of lymphatic contractions; and 2) the di-phosphorylation of MLC_20_ regulates the pressure-dependent changes in tonic contractions. The importance of the regulatory roles of MLC_20_ in lymphatic contractions was also confirmed by enhancing Rho-associated kinase (ROCK) (Kurtz et al., 2014) or by inhibiting ROCK (Hosaka et al., 2003; Si et al., 2013). ROCK increases calcium sensitization by phosphorylating the MYPT1 subunit of MLCP to inhibit its activity (Somlyo and Somlyo, 2004; Pang et al., 2005). Inhibition of MLCP shifts the kinase and phosphatase balance in favor of MLCK, thus a greater level of MLC_20_ phosphorylation is achieved at a given intracellular Ca^2+^ in the lymphatic vessel. Expectedly, inhibition of ROCK decreased lymphatic tone in the iliac collecting lymphatic vessels and thoracic duct (Hosaka et al., 2003; Si et al., 2013). In mesentery collecting lymphatic vessels, inhibiting ROCK decreased tone, contractile amplitude, and lymph flow while enhancing ROCK increased tone (Kurtz et al., 2014). The inhibition of MLCP is also mediated by direct binding and the inhibition of protein kinase C (PKC)-potentiated phosphatase inhibitor of 17 kDa (CPI17) (Somlyo and Somlyo, 2004; Pang et al., 2005; Berridge, 2008). When PKC was activated by phorbol ester, smooth muscle contraction was promoted independently of Ca^2+^ by MLCP inhibition (Jiang and Morgan, 1987, 1989; Masuo et al., 1994). Lymphatic vessels exhibited decreased Ca^2+^ sensitivity by reducing CPI-17 after β-escin permeabilization (Dougherty et al., 2008). A previous study showed that both MLC-dependent and MLC-independent mechanisms contribute to the Ca^2+^ sensitivity. Application of phorbol ester promoted MLC_20_ phosphorylation and an enhancement of tension of isolated permeabilized lymphatic, while later steady-state increased Ca^2+^ sensitivity independent of MLC_20_ phosphorylation (Dougherty et al., 2014). Conversely, activation of either PKA and PKG have noted dilatory effects on lymphatic muscle (Scallan et al., 2016), likely through inhibition of RhoA phosphorylation and suppression of ROCK activity (Murthy et al., 2003). High concentrations of caffeine (high μM -mM) also results in dilation of lymphatic vessels, typically after a transient large contraction driven by calcium release from RYRs (Jo et al., 2019). This is likely due in part to caffeine’s non-selective inhibition of phosphodiesterases (PDEs).

### Role of Endothelial Nitric Oxide Synthesis in Lymphatic Contractility

Nitric oxide (NO), a well-known vasodilator, is also an essential regulator of lymphatic pumping (Yokoyama and Ohhashi, 1993; von der Weid et al., 1996; von der Weid et al., 2001; Gasheva et al., 2006; Bohlen et al., 2009; Bohlen et al., 2011). The elevation of NO contributes to the subsequent relaxation of lymphatic vessels, especially after a contraction (Gashev et al., 2002; Gasheva et al., 2006). NO inhibition impaired relaxation during diastole, which further diminished refilling of the lymph pump and stroke volume. Exogenous NO decreases the lymphatic contractile frequency with increased passive lymph flow, indicating a negative chronotropic effect of NO (Gashev et al., 2002; Gasheva et al., 2006; Scallan and Davis, 2013). Inhibition of eNOS enhanced lymphatic contractile amplitude in rat mesenteric lymphatic with increased basal tone (Nagai et al., 2011). Enhanced wall shear stress activates eNOS in LECs to produce NO during lymphatic contractile cycles (Bohlen et al., 2009). LECs in lymphatic capillaries are exposed to unidirectional laminar flow, while LECs in collecting lymphatics experience oscillatory flow (Choi et al., 2017). Interestingly, the direction of flow does not affect the flow effect on reduced contractile frequency. However, lymphatic contractile amplitude inhibition due to increased flow is influenced by the direction of the flow (Gashev, 2008). Unidirectional flow exhibited a stronger negative inotropic effect than the oscillatory flow. And the chronotropic effect was observed prior to the ionotropic consequences of flow in rat collecting lymphatics (Gashev, 2008).

### Lymphatic contractile dysfunction in metabolic disease; distinct features of phasic and tonic contraction

Several studies consistently show a strong correlation between metabolic syndrome pathology and lymphatic dysfunction (Chakraborty et al., 2010; Zawieja et al., 2012; Weitman et al., 2013; Blum et al., 2014; Savetsky et al., 2014; Chakraborty et al., 2015; Scallan et al., 2015; Zawieja et al., 2016a; Zawieja et al., 2016b; Garcia Nores et al., 2016; Nitti et al., 2016; Lee et al., 2017; Lee et al., 2018; Lee et al., 2020). Recent clinical studies with patients at various stages of lymphedema patients reveal that excess body fat and obesity contribute the lymphatic dysfunction (Greene and Maclellan, 2013; Mehrara and Greene, 2014; Greene et al., 2015; Greene et al., 2020). In addition to its role in fluid and macromolecular homeostasis, the lymphatic system is the nexus of dietary fat and antigen transport as well as immune cell priming. Our laboratory first reported lymphatic contractile dysfunction in the diet-induced metabolic syndrome rat model (Zawieja et al., 2012) fed high-fructose feed. The reduction in spontaneous contractile activity has been subsequently observed *in vivo* in both murine high-fat diet induced models and genetic models of obesity, which typically display the nexus of ailments of metabolic syndrome (Weitman et al., 2013; Blum et al., 2014; Savetsky et al., 2014; Garcia Nores et al., 2016; Nitti et al., 2016). Despite the enormous investment to discover of the impaired lymphatic development in disease, little is known about the molecular mechanisms in lymphatic contractile dysfunction during metabolic disease. We have shown that metabolic syndrome exhibits collecting lymphatic remodeling, resulting in reduced load capabilities and impaired the intrinsic contractility required for maintaining proper lymph flow (Zawieja et al., 2012; Chakraborty et al., 2015; Zawieja et al., 2016b). Lymphatics from metabolic syndrome animals show negative chronotropy at all pressures, which contributes to the reduced intrinsic flow-generating capacity of the lymphatics. Perinodal collecting lymphatic vessels in metabolic syndrome animals are noticeably smaller in diameter than control counterparts under pressurized conditions (Zawieja et al., 2012; Zawieja et al., 2016b; Lee et al., 2020). While there is a regional difference in lymphatic contractile impairment in metabolic syndrome animals, both impaired lymphatic muscle and lymphatic endothelial cells contribute to lymphatic pumping activity (Zawieja et al., 2012; Blum et al., 2014; Zawieja et al., 2016a; Zawieja et al., 2016b; Lee et al., 2020). Additionally, inhibition of NOS activity by LNAME partially restored mesenteric lymphatic contraction and frequency in MetSyn animals (Zawieja et al., 2016b), and similarly, lymphatic function was restored with iNOS inhibition 1400 W in diet-induced mouse models of obesity (Torrisi et al., 2016). In leptin receptor-deficient mice (*db/db*), disrupted lymphatic endothelial barrier integrity due to impaired NO production and regulation also drove mesenteric lymphatic hyperpermeability (Scallan et al., 2015) which was ameliorated with the phosphodiesterase 3 inhibitor cilostamide or arginine supplementation. Similar impairments in NO production, eNOS signaling, and barrier regulation have been recapitulated *in vitro* (Lee et al., 2018) using high glucose supplementation. Additionally, LEC dysfunction in metabolic disease appears to be driven by excessive nitrosative stress due to the recruitment and accumulation of iNOS expressing inflammatory cells within the peri-lymphatic space (Rehal et al., 2020). Taken together, lymphatic dysfunction in collecting lymphatic vessels is associated with the impairment of both LMCs and LECs by aspects of both metabolic dysfunction and inflammation in collecting lymphatic vessels that contributes to the lymphatic dysfunction.

### Potential pharmacological targets for therapeutic interventions to ameliorate lymphatic contractile dysfunction in metabolic disease

#### ATP-Sensitive K^+^ (K_ATP_) Channel

In LMCs, as with most smooth muscle, K^+^ permeability is critical for resting membrane potential regulation (Ohhashi and Azuma, 1982; Telinius et al., 2014a) that governs the muscle excitability and contractile activity. Hyperpolarization by elevated K^+^ conductance can either overwhelm the depolarizing influence of inwards currents, such as those provided by Ano1, to prevent or slow the depolarization to the critical threshold, or with excessive K^+^ currents hyperpolarize the membrane out of the voltage window permissive for rhythmic oscillations (Mizuno et al., 1999; Mathias and von der Weid, 2013; Davis et al., 2020; Garner et al., 2021; Kim et al., 2021). Four different classes of K^+^ channels have been identified in LMCs: Ca^2+^ -activated K^+^ channels, volage-dependent K^+^ changes, ATP-sensitive K channels (K_ATP_), and inward rectifier K^+^. Among the four different classes of K^+^ channels, K_ATP_ channels are a significant mediator of the membrane potential in collecting lymphatic vessels (Telinius et al., 2014a) and have been linked to lymphatic contractile dysfunction in disease. K_ATP_ channels mediate lymphatic contractility by negatively regulating electrical excitability through hyperpolarization of the LMC membrane potential out of the window permissive to electrical oscillatory behavior. In guinea pigs, activated PKA and/or PKG in the lymphatic muscle increases K_ATP_ channel activity resulting in hyperpolarization and contractile cessation (Mathias and von der Weid, 2013; Lee et al., 2014). Pharmacological activation of K_ATP_ channels reduces the lymphatic contractile frequency as pinacidil, a K_ATP_ activator induces hyperpolarization (Telinius et al., 2014a). K_ATP_ channels appear to be partially or transiently active under the resting condition in lymphatic vessels as glibenclamide (FDA-approved K_ATP_ antagonist) increases the lymphatic contractile frequency in the human thoracic duct (Telinius et al., 2014a; Davis et al., 2020). Open K_ATP_ channels prevent action potential generation in lymphatic vessels resulting in phasic contraction cessation, and clinically relevant concentrations of K_ATP_ channel may impair *in vivo* lymphatic contractile function (Garner et al., 2021). A relatively small alteration of resting membrane potential (e.g., 4mV) through K_ATP_ modulation is enough to initiate lymphatic phasic contraction from quiescent vessels (Telinius et al., 2014a) or cease electrical and contractile activity (Davis et al., 2020), indicating the powerful role of K_ATP_ as a mediator lymphatic contractile dysfunction.

While the role of K_ATP_ channels in hypertension is inconclusive (Miyata et al., 1990; Furspan and Webb, 1993; Kitazono et al., 1993; Kam et al., 1994; Kawata et al., 1998; Kalliovalkama et al., 1999; Ghosh et al., 2004), K_ATP_ function appears to be decreased in obesity (Erdös et al., 2002; Irat et al., 2006; Hodnett et al., 2008; Lu et al., 2013) and diabetes (Bouchard et al., 1999; Miura et al., 2003; Kinoshita et al., 2006; Li et al., 2015) in blood vascular smooth muscle cells. However, the roles of K_ATP_ channels mediating lymphatic dysfunction under conditions of metabolic diseases have yet to be adequately addressed. Lymphatic contractile dysfunction was noted in the mesenteric collecting lymphatic vessels of guinea pigs with TNBS induced ileitis due to activated K_ATP_ channels (Wu et al., 2006; von der Weid et al., 2012; Mathias and von der Weid, 2013). Although, there is controversy as to whether NO signaling activates K_ATP_ channels in murine LMCs as a role for BK, but not K_ATP_, was recently identified in the NO-mediated contractile dysfunction (Kim et al., 2021). Nonetheless, targeting the potent inhibitory effect of K_ATP_ channels on phasic contraction of the lymphatic vessel would be an attractive therapeutic candidate for lymphatic dysfunction in metabolic disease. Notably, inhibiting K_ATP_ increased the lymphatic contractile frequency in mesenteric lymphatic from metabolic syndrome rats (Zawieja et al., 2016b). While these results point to a role for K_ATP_ channels partially mediating the lymphatic dysfunction observed in high fructose fed rats, whether it is a consistent mechanism of lymphatic dysfunction in obese murine models of lymphatic contractile dysfunction, and critically in human lymphatic vessel function, remains to be seen. Nor do these limited studies identify the signaling mechanisms upstream of, seemingly partial, K_ATP_ channel activation.

#### Sarcoplasmic Reticulum Ca^2+^ ATPase Pump, SERCA2a

While intracellular calcium has been considered a critical contractile regulator (Atchison et al., 1998; von der Weid et al., 2008; Dougherty et al., 2014; Lee et al., 2014; Zawieja et al., 2018), assessing the role of calcium release and uptake by the LMC SR as it pertains to lymphatic contractile regulation is still in its infancy. We have reported that MetSyn rats showed a significant reduction in lymphatic pumping due to decreased phasic contractile frequency and impaired intrinsic lymphatic muscle force production in skinned muscle preps where calcium is exogenously set. Since the activating voltage-dependent Ca^2+^ channels are indispensable for chronotropic and inotropic contraction of the lymphatics (Lee et al., 2014; Zawieja et al., 2018), we have examined the intracellular Ca^2+^ oscillation after activating voltage-dependent Ca^2+^ channels by depolarizing the insulin-resistant lymphatic muscle cell membrane with high exogenous potassium. The amplitude and peak cytosolic intracellular Ca^2+^ are not affected by insulin-resistant LMCs, suggesting voltage-dependent calcium channels are not diminished in metabolic diseased lymphatic muscles (unpublished data). We also found that insulin-resistant LMCs showed decreased endoplasmic reticulum calcium content without affecting non-sarcoplasmic reticulum Ca^2+^ ATPase (SERCA)-mediated calcium uptake. Inhibition of SERCA by the irreversible SERCA inhibitor, thapsigargin, in lymphatic vessels diminished both phasic contractile frequency and amplitude without affecting the tone (Lee et al., 2020). There are three distinct genes encoding SERCA pumps, SERCA 1, 2, and 3 resulting in more than 10 SERCA isoforms expressed in various muscles and non-muscle cells (Hovnanian, 2007). SERCA1 a and b are predominant isoforms in cardiac and skeletal muscle (Periasamy and Kalyanasundaram, 2007). Among SERCA2, SERCA2a is expressed in cardiac and type 1 slow-twitch skeletal muscle, while SERCA2b is predominantly expressed in smooth muscle cells (Hovnanian, 2007; Periasamy and Kalyanasundaram, 2007). Lymphatic muscle expresses both SERCA2a and SERCA2b isoforms (Lee et al., 2020), as lymphatic muscle exhibits a unique combination of muscle cell types that express both cardiac and smooth muscle contractile and regulatory proteins (Muthuchamy et al., 2003).

Metabolic syndrome condition decreased the levels of SERCA2a expression resulting in impaired Ca^2+^ regulation (Lee et al., 2020). SERCA2a is the predominant isoform in striated muscle, and the distinct smooth muscle isoform of SERCA2b is associated with phasic contraction in lymphatic vessels rather than lymphatic tone. Decreased SERCA activity has been observed in both cardiac and vascular tissues in mice with metabolic disease and decreased SERCA2a has been specifically linked to impaired cardiac contractility (Teshima et al., 2000; Trost et al., 2002; Hao et al., 2015). Type 1and type 2 diabetes models exhibit decreased SERCA2a expression in vascular smooth muscle (Belke et al., 2004; Wold et al., 2005). Notably, SERCA allosteric activator improves Ca^2+^ homeostasis both *in vivo* and *in vitro* metabolic disease models (Ferrandi et al., 2013; Kang et al., 2015; Singh et al., 2018). SERCA activation significantly improves the lymphatic contractile frequency in the isolated lymphatic vessels from MetSyn rats (Lee et al., 2020), suggesting SERCA2a as a therapeutic target in lymphatic contractile dysfunction.

#### eNOS

NO plays a critical role in the flow-mediated regulation of lymphatic contractility (Bohlen et al., 2009; Bohlen et al., 2011; Gasheva et al., 2013). With each contraction cycle, transient NO production is hypothesized to assist in the subsequent relaxation of lymphatic vessels and thus provide a lusitropic effect (Gashev et al., 2002; Gasheva et al., 2006; Bohlen et al., 2009). NO consistently exhibits a negative chronotropic effect, teleologically explained as reducing lymphatic vessel resistance under conditions that favor passive lymph flow (Gashev et al., 2002; Gasheva et al., 2006; Bohlen et al., 2009; Bohlen et al., 2011). Endothelial dysfunction in the blood vasculature has been well documented, particularly as it relates to reduce NO bioavailability. Unsurprisingly, the thoracic duct from MetSyn rats displayed reduced flow-mediated inhibition of contractility. Contraction frequency was particularly insensitive to imposed flow in MetSyn thoracic ducts, which was also associated with decreased eNOS expression (Zawieja et al., 2016a). In addition, MetSyn rats also show an impairment in insulin sensitivity with reduced eNOS phosphorylation and NOS production (Lee et al., 2018). Notably, inhibition of eNOS with generic NOS inhibitor (i.e., L-nitro-arginine methyl ester) abrogates the differences in the flow-mediated contractile frequency mediation (Zawieja et al., 2016a).

NO is also known as a critical regulator for endothelial barrier integrity. Studies have provided evidence that NO mediates lymphatic permeability (Kubes and Granger, 1992; Yuan et al., 1992; Yuan, 2006; Scallan and Huxley, 2010; Di Lorenzo et al., 2013; Scallan et al., 2013; Scallan et al., 2015). NO directly increased permeability in mouse collecting lymphatic vessels when directly assessed (Scallan et al., 2015). Diabetic mice exhibiting insulin-resistance increase permeability, and inducing NO synthesis decreases permeability (Scallan et al., 2015). Since the eNOS directly mediates both contractility and permeability in collecting lymphatic vessels impaired in metabolic disease, restoring lymphatic eNOS activity while inhibiting the aberrant NO production by activated immune cells (Torrisi et al., 2016) (Rehal et al., 2020) is an attractive pharmacological target in metabolic disease.

#### Vascular Endothelial Growth Factor Receptor (VEGFR) 3

Vascular endothelial growth factor receptor (VEGFR) 3 and its signaling cascade play a pivotal role in the development and normal function of the lymphatic system (Irrthum et al., 2000; Schulte-Merker et al., 2011). Mutations in the VEGFR3 (FLT4) gene in humans induces hereditary primary lymphedema. Milroy disease specifically, is an autosomal dominant congenital form of lymphedema that is typically attributed to mutations within VEGFR3 (Irrthum et al., 2000; Karkkainen et al., 2001; Schulte-Merker et al., 2011). The importance of VEGFC and VEGFR3 signaling in disease has been recently highlighted by Norden et al. (2020) (Norden and Kume, 2020). Although much is known about the role of VEGFR3 in lymphangiogenesis, less is understood regarding the role of VEGFR3 activation in physiological regulation in collecting lymphatic vessels. Notably, VEGF-C induced VEGFR3 activation in collecting lymphatic vessels resulted in a positive chronotropic response and elevated lymphatic vessel diameter, which was also associated with increased lymphangion stroke volume and lymphatic pumping output (Breslin et al., 2007). Since VEGFR3 is predominantly expressed on lymphatic endothelial cells in collecting lymphatic vessels, the VEGFR3-mediated changes in lymphatic pump activity are communicated from lymphatic endothelial cells to the lymphatic muscle cells. However, the VEGFC is also likely to act on myeloid cells that may express VEGFR3 and alter the inflammatory profile in a lymphatic endothelial independent fashion as well (Keller et al., 2021). As lymphatic muscle cells and lymphatic endothelial cell layers appear to be largely electrically isolated (Castorena-Gonzalez et al., 2018), impairments in VEGFR3 signaling in the LECs are likely to be communicated via paracrine signaling factors such as prostaglandins, NO, and other vasoactive compounds. The obese resistant mouse strains (BALB/cJ and MSTNln) maintained normal expression of VEGFR3 and normal lymph flow compared to obese prone mice (C57BL/6 J) when subjected to a high-fat diet (Garcia Nores et al., 2016). The study suggests the meaningful role of VEGFR3 signaling in the physiological lymphatic contractile function that protects against obesity. Notably, the collecting vessel plays an indispensable role in subcutaneous adipose tissue expansion and lymph transport homeostasis in obesity-induced lymphatic dysfunction, suggesting the importance of targeting collecting vessels in metabolic disease (Blum et al., 2014). K14-VEGFC mice that overexpress VEGF-C under control of the keratin-14 promoter in skin show an expanded dermal lymphatic vessel network and lymph draining dermal area. A high-fat diet decreases lymph transport in K14-VEGFC mice skin, suggesting the role of collecting lymphatic vessels as a key lymph transport machinery during the progression of obesity (Blum et al., 2014). Stimulating VEGFR3 signaling via its ligands (e.g., VEGFC or VEGFD) showed protective effects in metabolic disease (Savetsky et al., 2015; Chakraborty et al., 2019). However, Cao et al. (2021) showed a severe lymphatic architectural disorganization and excessive lymphatic collecting vessel branching and permeability using a diet-induced model of murine obesity with 15 and 32 weeks of high-fat diet (Cao et al., 2021). This was determined to arise as a consequence of Cox-2 dependent PGE2 formation which enhanced VEGFC production and VEGFR3 signaling. In this study, some lymphatic vessels were disrupted to the point that dye tracer could not be effectively transported past certain locations and was leaking into the mesenteric tissue, thus resulting in lymph accumulation in the mesenteric perivascular adipose and ultimately decreased insulin sensitivity (Cao et al., 2021). Surprisingly, this phenotype was not observed in the genetic db/db murine model of obesity for reasons that are unclear but are assumed to be high-fat diet-dependent and requiring long-term feeding (>15weeks).). Inactivating VEGFR3 disrupts triglyceride absorption in intestinal initial lymphatic, suggesting critical role of VEGFR3 signaling in fat absorption and retention (Shew et al., 2018). Thus, collecting lymphatic vessels appear to be a consistently and broadly affected by metabolic disease progression, and titrating the proper degree of VEGFR3 stimulation could be beneficial with synergetic effects as a therapeutic target between lymphatic network maintenance in initial lymphatics and promoting lymphatic contractility in collecting lymphatic.

## Conclusion and Future Perspectives

**FIGURE 1:**
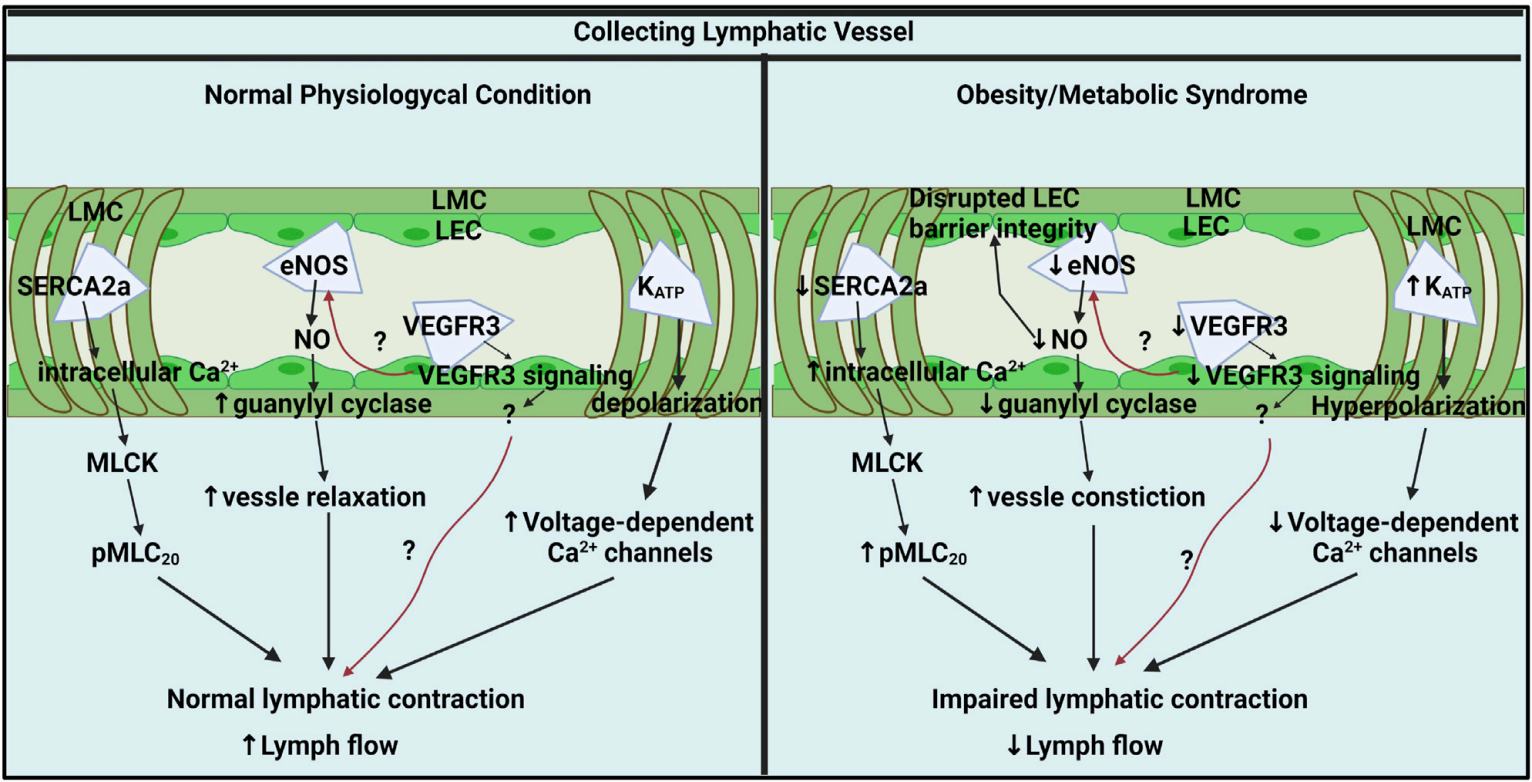
Key molecular pathways in collecting lymphatic vessel contractile activity under physiological and obesity/metabolic syndrome conditions. In normal physiological conditions, lymphatic contraction is critical for lymph flow modulation and regulated by intracellular Ca^2+^ in LMCs, NO from LECs, LMC membrane potential, and LEC VEGFR3 signaling. Ca^2+^ homeostasis is critical for lymphatic pump regulation and SERCA2a mediates intracellular Ca^2+^ by up taking intracellular Ca^2+^ back to SR. Intracellular Ca^2+^ stimulates MLCK and induces MLC_20_ phosphorylation, a critical lymphatic contractile regulatory protein. Flow-mediated NO from eNOS is an important lusitropic mediator in lymphatic contraction. VEGFR3 signaling via LECs might be a critical molecular signal that controls lymphatic contractility by directly impacting LMCs or indirectly mediating eNOS. K^+^ permeability is critical for resting membrane potential regulation that governs lymphatic muscle excitability and contractile activity. K_ATP_ channels mediate lymphatic contractility by negatively regulating electrical excitability. In metabolic syndrome, SERCA2a expression and activity are diminished that disturbing intracellular Ca^2+^ homeostatic resulting impairs lymphatic phasic contractions. Elevated intracellular Ca^2+^ promotes MLC_20_ phosphorylation via MLCK. Metabolic syndrome diminishes NO bioavailability by impairing LECs PI3K/AKT pathway. Decreased NO contributes to constricted vessels and impairs lymphatic contraction and lymph flow. VEGFR3 signaling pathways might be diminished in obese LECs that reduces lymphatic contraction directly impacting LMCs or indirectly via NO mediation. K_ATP_ channels are hyperactivated results in hyperpolarization of LMCs. The hyperpolarization by active K_ATP_ channels inhibits action potential generation in LMCs resulting in lymphatic contractile dysfunction in obesity. Generated with BioRender.com (Toronto, ON, Canada).

It is clear that the lymphatic collecting vessel and lymphatic vasculature are consistently and widely impacted by metabolic diseases, and lymphatic dysfunction serves as a nexus point that exacerbates metabolic disease progression. Despite considerable progress in understanding critical roles of the lymphatic system in metabolic diseases, many questions, especially in regard to the understanding of the pathophysiological mechanisms of lymphatic contractile function still remain unclear. There are only few studies that have investigated the molecular mechanisms and physiological role of lymphatic contractile function in obesity or other metabolic disorders (Table 1) Considering that the functional impairments of the collecting lymphatic vessels are predominant leads in metabolic diseases, understanding the molecular mechanisms in collecting lymphatic vessels in metabolic disease is necessary for the discovery of pharmacological targets to treat lymphatic dysfunction (Figure 1). The molecular cellular pathways that modulate lymphatic dysfunction in metabolic disease remain largely unknown both clinically and preclinically. Thus, while preclinical models display the molecular targets that mediate the positive chronotropic or inotropic function of lymphatic in metabolic disease, it remains to be discovered whether the lymphatic dysfunction can be reversed in patients with prolonged metabolic disorders from a therapeutic perspective. Understanding the cellular mechanisms that regulate lymphatic dysfunction in obesity and how lymphatic dysfunction can be resolved with targeted treatments from discovered molecular mechanisms will profoundly impact various metabolic diseases and provide therapeutic targets.

**TABLE 1:** Therapeutic targeting for collecting lymphatic function in obesity, diabetes, and metabolic syndrome.

Molecular target	Study model	Pharmacological/genetic intervention	Collecting lymphatic function	References
eNOS	High fructose induced metabolic syndrome rat	S-nitro-*N*-acetyl penicillamine (SNAP) L-nitro-arginine methyl ester (l-NAME)	↓ flow-mediated contractile frequency inhibition in MetSyn	Zawieja et al. (2016a)
			Inhibition of NOS abolished the differences in the shear-dependent contraction frequency between control and MetSyn	
	db/db mice	l-arginine L-nitro-arginine methyl ester (l-NAME)	↑permeability in mesenteric collecting lymphatic	Scallan et al. (2015)
			↓permeability in db/db collecting lymphatic by NO substrate	
SERCA	High fructose induced metabolic syndrome rat	Thapsigargin CDN1163	↓ Lymphatic phasic contraction in mesenteric collecting lymphatic in MetSyn	Lee et al. (2020)
			Inhibition of SERCA abolishes the difference in the phasic contraction between control and MetSyn	
			↑lymphatic contraction partially by activating SERCA in collecting lymphatic vessel	
VEGFR3	High-fat diet induced obese mice	K14-VEGFC	↓ collecting lymphatic vessel function in obese mice	Blum et al. (2014)
			↓ tracer spread in dermal lymphatic vessel in high-fat diet fed K14-VEGFC mice	
K_ATP_	High fructose induced metabolic syndrome rat	Glibenclamide	↓ Lymphatic phasic contraction in mesenteric collecting lymphatic in MetSyn	Zawieja et al. (2016b)
			↑ Lymphatic phasic contraction by inhibiting K_ATP_ channel in mesenteric collecting lymphatic	

## Abbreviations

ANO1, Calcium-activated chloride channel ANO1/TMEM16A; AP, action potential; BK, large-conductance calcium-activated potassium channels; COX2, Cyclooxygenase-2; eNOS, endothelial nitric oxide synthase; ER, endoplasmic reticulum; IP3R, Inositol 1,4,5- trisphosphate receptor; K_ATP_, ATP-sensitive K^+^channel; LEC, Lymphatic endothelial cell; LMC, Lymphatic muscle cell; MLC_20_, Myosin light chain 20; MLCK, Myosin light chain kinase; MLCP, Myosin light chain phosphatase; NO, Nitric oxide; PDE, Phosphodiesterase; PGE2, Prostaglandin E_2_; PKC, Protein kinase C; ROCK, Rho-associated protein kinase; RYR, Ryanodine receptor; SERCA, Sarcoendoplasmic reticulum calcium transport ATPase; SR, Sarcoplasmic reticulum; VEGF, Vascular growth factor; VEGFR, Vascular growth factor receptor.
